# Exploration of the definition and components of food and nutrition literacy among junior secondary school students: a qualitative study

**DOI:** 10.1186/s40795-022-00519-6

**Published:** 2022-03-30

**Authors:** Kingkaew Samruayruen, Nithra Kitreerawutiwong

**Affiliations:** Faculty of Public Health, Naresuan University, 65000 Phitsanulok, Thailand

**Keywords:** Food and nutrition literacy, Junior secondary school students, Qualitative study

## Abstract

**Background:**

Food and nutrition literacy (FANLit) has been proposed to promote healthy diet behaviour and is believed to decrease the prevalence of overweight, obesity and chronic disease. However, the perspective of junior secondary school students, aged 12–15 years and stakeholders towards FANLit is not well-defined. Thus, this study aims to explore the definition and components of FANLit.

**Methods:**

This qualitative study was carried out in Health region 2 including Phitsanulok, Sukhothai, Tak, Uttradit and Pethchboon. 49 participants from national and regional levels of Thailand were recruited. Data were collected by in-depth interview and focus group discussion using semi-structured questionnaire. Interview data were transcribed verbatim and analysed using thematic analysis.

**Results:**

FANLit is defined as an individual’s level of knowledge and skills (fundamental, interactive and critical) that relates to food and nutrition for appropriate selection of food and nutrient. Four themes of FANLit were identified as follows: 1) food and nutrition knowledge (Subthemes: food knowledge, food understanding, nutrition knowledge and nutrition understanding), 2) functional food and nutrition literacy (Subthemes: accessing to food and nutrition information, food selection, food preparation, daily food and nutrition planning and management), 3) interactive food and nutrition literacy (Subthemes: communication with others, sharing and discussion), and 4) critical food and nutrition literacy (Subthemes: information used in decision making, healthy food selection and effective budgeting).

**Conclusion:**

The results presented beneficial information regarding FANLit definition which is the initial stage to develop the assessing instrument and the intervention to improve FANlit in the adolescent group.

## Introduction

WHO [1] indicated that food consumption as one of modifiable factors that associate with chronic disease. The high-risk behaviour of unhealthy eating is related to the risk of overweight and obesity, type 2 diabetes mellitus (T2DM) and the metabolic syndrome. Therefore, shaping dietary behaviour is particularly essential in the chronic disease prevention [2, 3].

The term “health literacy” (HL) appeared in the first time in 1974 in a paper on minimum health education standards for all grade-school levels in the United States (US) [4]. It has received extensive attention in the publication in the 1992 National Assessment of Adult Literacy (NAAL) [5]. This term was widely accepted in the definition of World Health Organization (WHO), the American Medical Association (AMA), and the Institute of Medicine (IOM) in 1998, 1999, and 2004, respectively [6–8]. HL was developed from the concept of translating to practice into three forms, i.e. functional HL, interactive HL and critical HL [9]. Due to the board scope of health issue and concern in dietary which is relevant to food selection to meet nutritional requirements, the terms “food literacy” (FL) and “nutrition literacy” (NL) first appeared in a book published in 1992, and 1995, respectively [10–12]. While food provides energy, nutrients affect health [13]. The specific term “food and nutrition literacy” (FANLit) was introduced due to the complication of food environment that requires the improvement on specific knowledge and competence [12].

Previous literatures support that FANLit is a vital factor in improving the selection of food and maintenance of appropriate food pattern that links to nutrient delivery [14–16]. The investigation on FANLit could be used to design appropriate intervention and policies for specific group of concern [14]. Evidence showed a variety of definitions of food literacy [17, 18], nutrition literacy [19, 20], and food and nutrition literacy [12, 21]. Some studies attempted to describe FANlit with the methodology of systematic and literature reviews [12, 22]. However, other studies utilised the methods of instrument developed to explore FANlit in school-age and high-school graduates and adolescents [16, 21, 23]. Although the definition of food and nutrition literacy (FANLIt) was conducted in different contexts, this concept is limited in Thailand as only nutrition literacy [19] and food literacy were found [24]. As food is a complex combination of nutrients and other essential compounds for human health, adequate food consuming in the appropriate amount and correct proportion to meet the daily requirements of essential nutrients is related to nutritional status [13]. Therefore, FANLit have been identified as key component in the promotion and maintenance of healthy dietary practices [22].

Exploration on the definition of FANlit was conducted in different contexts, such as in educational curriculum, food culture, food marketing, political and social contexts, and various groups with different ages. However, FANlit definition was still limited in Thai context. The ages of 12 -15 years in junior high school student is the period of having independence in decision making on their food consumption. Providing a clear definition and its component on food and nutrition of this particular group could be beneficial in enhancing the cognitive function and promoting primary prevention of overweight and obesity, such as the development of FANLit intervention aligned with the Thai context. According to the aforementioned points, the multi-dimensional concept of FANLIt should be developed. This study aims to explore the definition and components of FANlit among junior secondary school students with the involvement of stakeholders from both national and regional levels of Thailand. A well-defined of FANlit could be useful in the designation of the instrument, intervention, and policy to improve their competency to achieve healthy dietary pattern and prevention of diet-related noncommunicable diseases.

## Methods

This study was conducted using qualitative approach for clarification of FANLit definition which may be used to develop a tool for assessing FANLit competency to improve their competence. For the interpretivist paradigm, all knowledge is induced from particular experience. The stakeholders in this context were unique with rich and valuable knowledge and ideas which were shown to be influential [25]. Data collection took place for a year (between May 2019 to May 2020). The participants were informed regarding the research aim, information confidentiality, and right to revoke the participation without punishment. The junior high school students obtained the assent from their parents or guardians. The researcher provided the participants information sheet and assent form, ensured their understanding towards them gave the opportunity for enquiry, and explained for anything unclear. The written informed consent was provided to all participants for their voluntary participations.

### Setting

This study was carried out in Sukhothai, located in lower Northern, Thailand. This province had a high prevalence of overweight and obesity in Health region 2 (Health region 2 includes Phitsanulok, Sukhothai, Tak, Uttradit and Pethchboon). In 2018 – 2021, the prevalence of overweight among the children aged 6–14 years old was 6.98, 6.69, 9.90 and 10.08 whereas the prevalence of obesity was 9.08, 8.23, 6.52 and 6.60, respectively [26]. The policy makers, practitioners (nurses and school health teachers), and students at district and provincial levels were purposively drawn from these provinces. However, to analyse the linkage of perspectives in national and community levels, the policy makers and academics were selected at the national level.

### Recruitment and sampling

Purposive sampling was used to select participants. They were divided into four groups: 1) policy makers, 2) practitioners (nurses, school health teachers), 3) academics with experience on food and nutrition for both provincial and national levels, and 4) junior secondary school students with normal weight and overweight or obesity. The data collection was considered as bottom-up approach as the perspectives from the students were gained, together with relevant inductive approach which its results were emerged from the key informants. The criteria of the eligibility requirements for the participants who were the key authorities responsible for food and nutrition at national, provincial, and district levels included the following: 1) being policy makers at provincial and national levels, practitioners at district and provincial levels, academics are nutrition experts at the provincial and national levels, or students at provincial level; 2) allowing data collection to take place onsite; and 3) willing to participate in the in-depth interview or FGDs.

Participants were contacted by direct contact (via telephone). The first author recruited the participants according to the protocol. The appointment and consent form were delivered via email. KS and NK conducted the in-depth interview and focus group discussion (FGD). KS and NK concurrently analysed the data while collecting the data to ensure that no new issues were emerged during the interviews with all 49 participants (Table 1).

**Table 1:** Characteristics of the participants

Level of participant	Position	Age (Years)	Gender	Date of interview	Type of Data collection	
					**In–depth interview:** **Number of participants**	**FGD:** **Number of participant (Group number)**
National level	Policy maker (Responsible for food and nutrition policy, working at Ministry of Public Health National Health Security Office)	56	Female	14 May 2019	1	
	Academic 1 (Nutrition expert)	51	Female	15 May 2019	1	
	Academic 2 (Nutrition expert)	56	Male	21 May 2019	1	
	Academic 3 (Nutrition expert)	56	Female	29 May 2019	1	
Provincial level	Policy maker (Responsible for food and nutrition policy, working at Provincial Medical Health Office)	52	Male	22 July 2019	1	
District level	Policy maker 1 (Responsible for food and nutrition policy, working at District Health Office)	41	Male	18 June 2019	1	
	Practitioner 1 (Director of general hospital and community hospital)	53	Female	20 Aug 2019	1	
	Practitioner 2 (Director of general hospital and community hospital)	39	Male	5 July 2019	1	
	Practitioner 3 (Director of general hospital and community hospital)	46	Male	10 June 2019	1	
	Practitioner 4 (Director of general hospital and community hospital)	59	Male	17 July 2019	1	
	Practitioner 1 (Nurse at community hospital and sub district health promotion hospital)	34	Female	30 July 2019	1	
	Practitioner 2 (Nurse at community hospital and sub district health promotion hospital)	44	Female	31 July 2019	1	
	Practitioner 3 (Nurse at community hospital and sub district health promotion hospital)	35	Female	7 Aug 2019	1	
	Practitioner 1 (School health Nurse, responsible for food and nutrition of the student)	58	Female	8 Aug 2019	1	
	Practitioner 2 (School health Nurse, responsible for food and nutrition of the student)	58	Female	8 Aug 2019	1	
	Practitioner 3 (School health Nurse, responsible for food and nutrition of the student)	46	Female	9 Aug 2019	1	
	Junior secondary school student 1	13	Female	3 Sep 2019	1	
	Junior secondary school student 2	13	Female	3 Sep 2019	1	
	Junior secondary school student 3	13	Female	3 Sep 2019	1	
	Junior secondary school student 4	14	Male	4 Sep 2019	1	
	Junior secondary school student 5	14	Male	4 Sep 2019	1	
	Junior secondary school student 6	15	Female	4 Sep 2019	1	
	Junior secondary school student 7	15	Female	10 Sep 2019	1	
	Junior secondary school student 8	14	Female	10 Sep 2019	1	
	Junior secondary school student 9	14	Female	10 Sep 2019	1	
District level	Practitioner (Nutritionist or nurses of general hospital and community hospital)	36, 43, 31, 42	Female	11 Mar 2020		4 (Group no.1)
	Practitioner (Nurse at community hospital and sub district health promotion hospital)	30,48,32, 42,27,50	Female	26 Feb 2020		6 (Group no.2)
	Practitioner (School health Nurse, responsible for food and nutrition of the student)	40,33,28, 26,34,37	Female	19 Feb 2020		6 (Group no.3)
	Junior secondary school student	13,13, 15,15	Female	5 Feb 2020		4 (Group no.4)
	Junior secondary school student	13, 13, 14,14	Female	12 Feb 2020		4 (Group no.5)
**Total of participants**					**25**	**24 (5 FGDs)**

### Data collection

The in-depth interview and focus group discussion were performed in accordance with the guideline of semi-structure interview. Prior to the interview, KS telephoned the participants to make an appointment with them according to their time availability and provide them the research information. The participants who met the criteria were invited to participate. The researcher explained the objectives of the research, the contribution, methods and duration, and risk and benefit of participating in this study. The participants were asked regarding their clarity and comprehension for this voluntary participation. The written informed consent was obtained from all participants. As the permission from parents and guardian was required when carrying out research on students with the age below 18 years old, the informed parental written consent and assent form of the student were completed before the participation. In-depth interview allowed the interviewer to deeply explore the respondent’s feelings and perspectives towards the definition and determinants of FANLit. The interviewing process lasted for 30 -60 min (Median = 60 min, Minimum = 30, Maximum = 60) [27, 28]. In addition, FGD was carried out to discuss specific topics with the aim to draw complex personal experience, belief, perception and attitude of the participants through moderated interaction [29]. This lasted 60–80 min (Median = 70 min, Minimum = 60, Maximum = 80). Data collection had been undertaken until reaching data saturation.

All researchers were trained regarding qualitative research methods, including research ethics, basic qualitative research methods, data collection procedures, data analysis and interviewing experience with data collectors and coders. The semi-structure interview guideline was developed by KS and proofread by NK, using the health literacy framework which is the broad concept to guide the questions to explore the meaning and components of specific areas of food and nutrition. This methodology consonant with Eley C et al. [30]. The open-ended questions were created with the key questions and probe questions. The questions allowed further exploration towards the attitudes of the participants and strengthened the content. The questions were composed of the definition, knowledge and skill regarding food and nutrition, and the components of food and nutrition literacy. The semi-structure interview guideline was pre-tested with five participants who were the public health authority working in provincial medical office, a nurse working in primary care facility, a school health nurse, a student, and a lecturer with experience in food and nutrition science. The pilot study resulted in minor revision on the clarification of the questions. The interview questions are in Table 2. Before the interview, the permission for the audio-recording during interview was approved and the consent process was completed by the participants.

**Table 2:** Key questions for semi-structured interviews guideline

List of questions
1. What is the definition of health literacy?
2. What are the definitions of food literacy and nutrition literacy? Are there any differences between these two terms?
3. What are the benefits of those with health regarding food and nutrition?
4. When should we begin to raise awareness among the public regarding health literacy on food and nutrition?
5. What do food knowledge and nutrition knowledge mean, and how do we assess them (food and nutrition knowledge)?
6. What do food skill and nutrition skill mean, and how do we assess them (food and nutrition skills)?
7. What components should be included in the health literacy on food and nutrition among junior secondary school students?
8. Any issues that are missed or issues that you would like to raise regarding food and nutrition literacy?

### Data analysis

All interviews were carried out by KS and NK in February 2019. The interview was transcribed verbatim. The verbatim transcription was delivered to researchers for proofreading. The data were manually analysed through content analysis. KS and NK performed inductive coding via independently reading the transcripts to elaborate the code from the data set delivered from the field [31]. The two codes were compared by the researcher and theory-informed. The discrepancy code was discussed until consensus was reached. The examples of inductive code from the field are "Food Knowledge", "Food understanding", "Nutrition knowledge", "Nutrition understanding", "Access to food and nutrition information", "Sharing and discussion regarding health and nutrition with others" and "Evaluate information about food and nutrition". The next step of data analysis was independently presenting data in theme and subtheme. The second meeting was held for data analysis and discussion of the result. The next interview was prepared through the list of questions that should be added for the next participants to probe the questions, confirm, or verify some issues. The analysis was performed in parallel with the data collection and replication from the interviews.

### Trustworthiness

Credibility was reached through audio recording and verbatim transcription of all interviews. This is to ensure that the findings represented the information drawn from the participants’ original data in the field. Additionally, to create familiarity with the setting and context, and to build trust to deliver factual data according to the time planned for data collection as mentioned, credibility of the prolonged engagement was carried out during the interview with appropriate time. Member checking was conducted by asking for feedback on the interviewing issue at the end of the interview through summarization of field notes [32]. Method triangulation was achieved by utilising in-depth interview and FGDs in data collection. Person triangulation was performed by the key informants, including policy makers, practitioners, academics and the adolescents to gain all perspectives that might be relevant to the specific issues of FANlit. Transferability was implemented through thick description of experiences and contexts that could be applied to other contexts [33, 34].

## Results

A total number of 49 participants were interviewed with three policy makers, 26 practitioners, three academics and 17 adolescents. The definition and components of FANLit were presented in Table 3, together with four themes, thirteen subthemes and quotes.

**Table 3:** Overview of the themes and subthemes with quotations of FANLIt

Theme	Subtheme	Quotation example
**Theme 1** **Cognitive: Food and nutrition knowledge** Knowledge and understanding on meaning, types, components and food sources, Thai healthy index, food safety, amount, sources and functions of nutrition, food label, and effects of food and nutrition on health		“It is knowledge and understanding of food and nutrition on safe food consumption which is beneficial to the body”. (IDPch_3_) “Students should be able to identify types of food and food safety. They should be able to tell us nutrients of the food their consumed, and the extent they are beneficial to our body. They should also read food label.”. (IDP_3_)
	**Subtheme: Food knowledge** Fundamental knowledge on food, including definition, types, ingredients, contaminants, sources of food and food-based dietary guidelines	“For the first issue, the literacy of food should be composed of definition, physical components of food, types, sources, knowledge and extent regarding types of nutrients for children at this age, according to food-based dietary guidelines.” (IDPo_2_)
	**Subtheme: Food Understanding** Understanding the thinking and utilising fundamental concept on food, including safe food and effects of food on health	“When students can explain that our health is related to food we consumed, such as mentioning that excessive sugar intake might cause diabetes in the future…” (IDP_3_)
	**Subtheme: Nutrition knowledge** Fundamental knowledge of nutrition, including types, amount, sources, functions of nutrients and knowledge in reading nutrition facts	“Children understand that food is fundamentally necessary for our health, such as strengthening our bodies, increasing immunity with less chance of having illness and infection. It can also remedy illness.” (IDPo_2_)
		“Nutrition literacy are knowledge on functions of nutrients, including creating and mending cells that affect the working of our bodies.” (IDPo_2_)
		“Children should be educated on nutrition, including types of nutrients, amount of nutrients they should get, sources of nutrients and nutrition information on food label. “ (FGPt_4_)
	**Subtheme: Nutrition understanding** Understanding the thinking and utilising fundamental knowledge regarding food nutrition, nutrition fact label and effects of nutrition on health	“Students should understand nutrients that are necessary for our bodies, and how the amount of the nutrients affects our bodies. They should also know the meaning of nutrition flag.” (FGDPt_2_)
		“The meaning of food and nutrition literary is deeper than knowledge. It refers to not only having knowledge regarding food and nutrition, but also having understanding about them.” (IDPo_3_)
		“…accessing, understanding and utilizing. They should also understand food and nutrition to make accurate decision.” (IDA_2_)
		“The main focus is on health literacy, but the content is related to food and nutrition. Food and nutrition are the context to evaluate problems and issues. The actions of accessing, understanding and evaluating issues regarding food and nutrition of children should be continuous.” (IDA_3_)
		“Children should understand food information on the nutrition label to select appropriate food.” (FGPt_1_)
		““They should understand types of nutrients that strengthen their bodies, and know types of nutrients that may cause illness.” (FGPch_1_)
**Theme 2** **Skill: Functional food and nutrition literacy** Food and nutrition information accessing, selection, preparation, cooking and planning of daily food and nutrition		“Food and nutrition literacy is defined as the ability to have intellectual and social skills to search for information from sources and understand all information.” (IDPo_1_)
	**Subtheme: Accessing to food and nutrition information** Ability to search, ask for information on food and nutrition from sources, such as media and people	“Intellectual and social skills to search for information from sources for adolescents are defined as the ability to search for information to deal with health. They should also able to do the searching in English via the Internet and digital form.” (IDPo_1_)
	**Subtheme: Food selection** Ability to select both fresh and ready-to-eat food which is high quality and beneficial for our health	“Students have knowledge on type, price, quality, freshness of food to make a selection of appropriate food for themselves.” (IDPo_1_)
		“They should have accurate knowledge and should be aware of food that is beneficial and contaminant-free and organic, and utilise such skills to select, prepare and cook actual food.” (IDPo_3_)
	**Subtheme: Food preparation skills** Ability to prepare and cook, including food preparing, cooking healthy food, using cooking utensils, and food preserving	“Cleanliness and food preservation should be maintained, such as storing leftovers in the fridge, and heating up before having it. Meat should be stored in the freezer or being sun-dried.” (IDPt_5_)
		“It is the ability to cook various food, such as steaming, grilling, barbecuing, boiling or stir-frying.” (FGDPch_1_)
		“It is the ability to prioritize importance when dealing with their own food.” (IDPo_2_)
		“It is planning and aiming for having healthy food. They should also realise that each group of food has different essences to our health, resulting in different amounts of their intakes.” (IDPhph_1_)
**Theme 3** **Skill: Interactive food and nutrition literacy** Informing, inquiring, asking for advice, communicating, information exchanging, discussing food and nutrition with peers, family, academics, healthcare providers or other people, giving advice and convincing others to consume healthy food		“Food and nutrition literacy for adolescents includes interactive and critical levels. They should search for more information, i.e. they should be brave to ask questions.” (IDPo_1_)
		“Communication and information exchanging between themselves and others is important as they could think and analyze before making a decision on the practice of the food and nutrition.” (IDPo_2_)
	**Subtheme: Communication with others (e.g., peers, family, academics, healthcare providers) on food and nutrition** Ability to communicate, inform, inquire or ask for advice regarding food and nutrition from others	“It is the ability to communicate, access, and gain knowledge from service providers. Eventually, understanding the thinking process, analysing, categorising, synthesising and evaluating are the processes of creating new knowledge. We should be aware and could apply this knowledge to prevent illness. We should also be able to solve problem that might arise in the future.” (IDA_1_)
		“They should have sources where advice could be given, such as health mentor who can deal with food and nutrition.” (IDPo_1_)
		“We can ask advice from health education teachers, or doctors with specialization in nutrition.” (FGPch_1_)
	**Subtheme: Sharing and discussing regarding food and nutrition with others** Ability to exchange information, discuss, express opinion regarding food and nutrition, give advice and convince others to consume healthy food	“We may need skill to deal with our own health, and could accurately pass on this skill at individual level, and increase health quality.” (IDPo_2_)
		“Knowledge and understanding food and nutrition regarding safety of food consumption might be beneficial to our health, and might change consuming behaviour that is suitable for our bodies. We can also give others advice.” (IDPch_3_)
**Theme 4** **Skill: Critical food and nutrition literacy** Ability to evaluate and select information regarding food and nutrition, apply such information to make a decision on food consumption according to food-based dietary guidelines, deal with food budgeting, including expense management and comparison on food price when consuming healthy food		“After the students receiving information, they should be able to tell which information is accurate. Besides accessing information from all sources, they should be able to filter, examine and understand food and nutrition. This should result in accurate decision when selecting food to consume. The practice is important as food and nutrition literacy has the main focus on the implementation.” (IDA_2_)
		“Food and nutrition literacy in adolescents includes interactive and critical levels. When in doubt, they should ask question and argue which might lead to the change in behavior.” (IDPo_1_)
	**Subtheme: Information utilising in decision making** Ability to examine and analyse to evaluate and select information regarding food and nutrition from sources to achieve the aim of healthy food consumption	“Critical literacy refers to an ability to filter and select information from media, and evaluate it. At the moment, we have various sources of information on food on social media. This affects the decision making on food selection of people.” (IDPo_2_)
		“I wholeheartedly agree with decision making as it is related to the practice in thinking and examining. This might yield wisdom to believe in the message.” (IDA_2_)
		“It is a matter of intelligence, starting from accessing, understanding, analyzing, synthezing, evaluating and applying.” (IDA_3_)
		“When there is load of information, I will select the information by reading the websites, comparing them, and analyzing the possibility of it. This should help me make a decision on believing in them.” (IDS_9_)
	**Subtheme: Healthy food selection** Ability to make a decision to select healthy food	“Food literacy concerns the literacy in selecting types, price, quality and freshness in terms of food safety and hygiene. The last issue is accurate decision that leads to the practice on food selection.” (IDPo_1_)
		“It is the ability to search and study information that is necessary in food we are taking. It is also the ability to realize the extent the food is beneficial for our health and make a decision on food that is appropriate for our health.” (IDA_4_)
		“It is the ability to select and make a decision to buy and have food by reading nutrition label. It is also the ability to read the amount of sugar, sodium and fat to provide energy and benefits to our health.” (IDPch_1_)
	**Subtheme: Effective budgeting** Ability to manage costs on food, including expense management, and comparison on food that is healthy and worth consuming	“It is the effort to select food that is healthy although it might be more expensive than other food, or the effort to buy healthy food despite limited budget.” (IDP_4_)
		“The scope of health knowledge regarding food includes pricing and cost of food consumption, i.e. the food is worth buying, appropriate and healthy.” IDPo_2_
		“In selecting food, we need to consider whether it is fresh. For some food, getting it from market might be cheaper and fresher. I will also consider if it is contaminant-free, then compare the prices.” (FGDSn_2_)

### Definition of FANLIt

Participants had impression that FANLit was the abilities of individual, including knowledge and information accessing, thinking, communicating, information analysing, and decision making and budget preparing to manage food and nutrition. Food was relevant to preparation and selection whereas nutrition was connected to nutrients that affected health.

“The definition of their literacy is the ability to have intellectual and cognitive skills. They should understand all information. Regarding food omniscience, children should not only know nutrition and the five-food groups, but they should also know the food that is suitable for them, and their health status.” [IDPo1]

“In terms of food literacy, food is about preparation, eating selection, information accessing, food and nutrition. This is relevant to the functions of the body, specific illness. Which level of the literacy do I have? There are three levels: fundamental, interactive and utilising. Level 1 is related to the literary on food and nutrition. Level two is understanding, thinking, evaluating, filtering, analysing and accurately synthesising message of the media. Level 3 is skills to be used in real life.” [IDPo2]

“A person with health literacy must have abilities and skills.” [IDA2]

“We should focus on health literacy, but selectively think of food and nutrition. Food and nutrition are the context to evaluate issues on food and nutrition of children. The definition of health literacy should be stated as health literacy on food and nutrition. It is the expression of children who expose to media, understand, read food labels, and take information regarding food and nutrition from talking with their peers, parents and teachers. The school curriculum provides the teaching on food selection, food preparation, cooking appropriate food to their ages, and planning to buy food. This allows children to expose, understand and evaluate accessed information on food and nutrition.” [IDA_3_].

In summary, FANLit was defined as the degree of knowledge and understanding, functioning, interactive and critical skills in preparing, cooking, selecting food, having accessibility and ability to share food information, and to access nutrition information to make appropriate nutritional decisions at individual level.

### The components of food and nutrition literacy

Table 3 provides the components of FANlit in four themes with illustrative quotes. Interview and focus group discussion resulted in four themes: 1) Cognitive: Food and nutrition knowledge, 2) Skill: Functional food and nutrition literacy, 3) Skill: Interactive food and nutrition literacy, and 4) Skill: Critical food and nutrition literacy.

In conclusion, food and nutrition literacy comprises cognition and skills that should be responsible by all stakeholders of junior secondary school students. The cognition is the process of acquiring knowledge and understanding through thinking, and its assessment should be performed by measuring knowledge and understanding specific issue. Additionally, skills are categorised as functional, interactive and critical on food and nutrition literacy. This study described functional literacy as the cognition on food and nutrition knowledge and ability to access food and nutrition information, select food, prepare food and cook, and plan daily food and nutrition. Interactive food and nutrition literacy is the ability to explain, inquire, or ask for advice, communicate, share knowledge, discuss food and nutrition with friends, family, academics, healthcare providers or other individuals, and advise or convince other people to consume healthy food. Lastly, critical food and nutrition literacy is the ability to evaluate and select information regarding food and nutrition, utilise such information and knowledge to make a decision according to food-base dietary guidelines and budget management. This ability includes expense management and cost-effective comparison on food price when consuming healthy food.

## Discussion

The objective to explore the perspective of all stakeholders towards the definition of FANLit has been achieved with the qualitative bottom-up approach. The concept of FANLit in this study was composed of cognitive knowledge, fundamental, interactive, and critical skills related to food and nutrition. This is useful for the decision on healthy food consumption. This concept is consonant with Bloom's taxonomy which indicated that knowledge is the fundamental cognitive skill and refers to the retention of specific and discrete pieces of information, such as facts and definitions, or methodology. Using knowledge, comprehending and utilizing skills or techniques in new situations through the application in the third level of Bloom’s taxonomy of learners are a manner of practice and maintenance to behave in the timely manner [35]. The definition of FANLIt with cognitive knowledge, functional, interactive, and critical skills was consistent with the previous literatures and Nutbeam’s model of health literacy [10, 14, 16, 23]. These dimensions are essential as they might influence the health of adolescents and reduce risks of developing diseases in the future, especially non-communicable diseases.

To be specific, the first theme of FANLit was cognition of food and nutrition knowledge with food and nutrition knowledge, and understanding in food and nutrition. This theme indicates that knowledge is the initial level of cognitive behaviour [35]. It also shows that understanding is the representation of knowledge. Therefore, knowledge and understanding should be included in the definition of FANLit. This is in agreement with previous literatures indicating that health literacy had a set of knowledge and skills or a hierarchy of functions in the definition [36, 37]. In the same way, the academics agreed to have a set of knowledge and understanding in the definition of FANLit [14, 23, 24, 36–39]. This concept could be linked to translate knowledge to practice which is corresponding to procedural knowledge and judgement skills in food and nutrition [40]. The content of knowledge and understanding of food and nutrition comprises definition, types, components and sources of food, nutrition, food safety, quantity, sources, functions of nutrients, nutritional fact label and the effects of food and nutrients on health. This content is in harmony with declarative knowledge that represents understanding of factual information about food and nutrition whereas procedural knowledge represents understanding of nutrition flag that influences reasonable choices and actions of food consumption [41]. This is the fundamental knowledge to interpret various issues of food and nutrition to establish suitable food consumption practice for one's own health.

The second theme of FANLit was a set of skills of functional food and nutrition literacy with the access to food and nutrition information, food selection and food preparation skills. This theme stipulated that FANLit was a basic skill in translating knowledge and understanding to the application in accessing information, selection and preparation of food and nutrition. This result is consonant with Nutbeam [9] proposing the three-level model of functional health literacy, interactive health literacy and critical health literacy. The findings of a study by Ashoori M, et al. [23] are similar to this study in that the majority of functional skills is related to the application of basic food and nutrition knowledge, shopping and storing foods, meal preparation skill, food production, and environmental sustainability skills. In line with the previous studies, accessing food and nutrition, information, food selection [38, 42, 43], food preparation skill [24, 38, 39],and daily food and nutrition planning and management [42, 43] are included in FANLit definition.

The third theme of FANLit was a set of skills of interactive food and nutrition literacy with communication, sharing and discussion regarding health and nutrition with others (peers, family, academics, healthcare providers, and so on). This theme explained that food consumption was daily activity of people, and most students living with their parents or guardians. It is also an interaction with their peers and teachers at school. Therefore, social skills of this group to communicate and share information on food and nutrition had been developed. This result is consonant with food and nutrition [20, 24, 38], knowledge-sharing and discussion on topics related to health and nutrition with others [38]. When the interactive skill of FANLIt is achieved, the application of new information will lead to the change in their practice and behaviour. The interrelation of functional skill to interactive skill in FANLIt is the ability to combine food and nutrition information with interactive social skill to adapt ourselves to new situation.

The fourth theme of FANLit was a set of skills of critical food and nutrition literacy with information used in decision making, healthy food selection and healthy budgeting. This theme indicated that this skill is the advance of translating factual knowledge, procedural knowledge, and social skill to practice, such as preparing budget to buy healthy food. This information is shown in the reply of the participants that several social media influenced decision making on food consumption. Similar to previous studies, these components affect healthy food selection [24, 38, 39] and effective budgeting [42, 43].

In this study, there are four themes and 12 subthemes in cognitive knowledge and skills. In regards to skill domain, it is in line with Nutbeam's hierarchical model of health literacy, including functional, interactive and critical literacy [9]. The perspectives from various types of stakeholders were different due to their roles, responsibilities and perception. For example, while the students reflected understanding on the skill in food preparation, and communication with the teachers and healthcare providers, school health nurses reflected understanding on the nutrition flag, and public health practitioners on the nutrient information and the skill in food selection. This information is complementary with the others perspectives in defining FANLIt and its component from all stakeholders. These findings could be beneficial for the development on assessing instrument for FANLit in the junior secondary school students. The findings on FANLit are essential as dietary pattern relates to daily living activities. Understanding FANLit might increase the cognitive knowledge, functional, interactive, and critical skills which leads to better management on their food choice, interaction with others on food and nutrition information and critical decision making on food consumption for their own health.

The use of qualitative approach to explore and understand the issue of FANLit is considered the strength of this study. Various perspectives from national and regional levels were encompassed. Nonetheless, the specific context of the subjects of this study could be regarded as limitation. Findings from this study could be useful in underpinning further development of assessing instrument of FANLit among junior secondary school students in Thailand. In addition, the results could be used as a guideline for future programs aiming at improving food and dietary behaviour in young adults.

## Conclusion

In regards to the definition and components of FANLIt in the context of Thailand, they could be divided into four themes and 12 subthemes. The results from this study suggest that FANLit has played an important role in understanding towards food and nutrition among the junior secondary school students. The four themes indicate the factors inducing eating decision in the adolescent group. They also point out the relationship between food and nutrition knowledge and skills which suggests the concurrent improvement of these. For future research, the research instrument on assessing FANLIt should be developed to improve the programme regarding the food and nutrition practice among the junior secondary school students.

## Data Availability

The dataset of this study is a message from the participants that was protected under the terms of Naresuan University Ethical Committee for disseminating. Additionally, Thailand’s Official Information Act 1997 prohibits the release of identifiable data relating to public bodies. According to the above limitations, interested parties may approach the correspondence to contact the secretary of Naresuan University Research Ethics Committee to supply further information hence the datasets generated and/or analyzed during the current study are not publicly available but are available from the corresponding author on reasonable request.
